# ﻿*Mycetia saxicola* (Rubiaceae), a new species with cauliflory from limestone areas in Yunnan, China, supported by morphological and molecular data

**DOI:** 10.3897/phytokeys.267.175218

**Published:** 2025-12-08

**Authors:** Zhuqiu Song, Buyun Zhang, Xiaokai Xiong, Dongxian Xu

**Affiliations:** 1 Key Laboratory of National Forestry and Grassland Administration on Plant Conservation and Utilization in Southern China, South China Botanical Garden, Chinese Academy of Sciences, Guangzhou 510650, China South China Botanical Garden, Chinese Academy of Sciences Guangzhou China; 2 Guangdong Academy of Forestry, Guangzhou 510520, China Guangdong Academy of Forestry Guangzhou China

**Keywords:** Argostemmateae, cauliflory, limestone, *
Mycetia
*, taxonomy

## Abstract

A new species exhibiting cauliflory within the tribe Argostemmateae (Rubiaceae), *Mycetia saxicola* Z.Q. Song & D.X. Xu, is described and illustrated from limestone areas in Yunnan, China. Our comprehensive study, which includes literature reviews, field and herbarium morphological observations, and molecular phylogenetic analyses of five plastid DNA regions, reveals that *M. saxicola* is closely related to *M. fangii* K.J. Yan & Z.Q. Song. Morphologically, both species share several key features, including cauliflorous inflorescences, few flowers per inflorescence, eglandular bracts and bracteoles, short corolla tubes, and calyx lobes much shorter than the corolla. However, *M. saxicola* can be readily distinguished from *M. fangii* by its limestone habitat, subcoriaceous leaves, linear stipules, very short pedicels and peduncles, and a calyx bearing two sessile, deciduous glands on each sinus. Additionally, this paper provides illustrations of six other relevant species of *Mycetia* to facilitate further taxonomic comparisons.

## ﻿Introduction

The coffee family (Rubiaceae) is the fourth-largest lineage of flowering plants, encompassing 586–615 genera and 14,181–14,266 species, which are classified into two subfamilies and 72 tribes (Razafimandimbison and Rydin 2024a, b; Verstraete et al. 2025). Among these, the subfamily Rubioideae comprises 30 tribes (Razafimandimbison and Rydin 2024a; Verstraete et al. 2025), one of which is Argostemmateae Bremek. ex Verdc. This tribe was validly established by Verdcourt (1958) based on morphological evidence and was subsequently redefined by Rydin et al. (2009), Ginter et al. (2015), Razafimandimbison and Rydin (2019), and Thureborn et al. (2022) using molecular data. The current concept of Argostemmateae is primarily supported by molecular evidence but appears to lack a clear morphological synapomorphy (Razafimandimbison and Rydin 2024a). Argostemmateae currently includes six genera: *Argostemma* Wall., *Clarkella* Hook.f., *Leptomischus* Drake, *Mouretia* Pit., *Mycetia* Reinw., and *Neohymenopogon* Bennet (Razafimandimbison and Rydin 2024a).

Historically, *Myrioneuron* R.Br. ex Hook.f. was accepted as a distinct genus by most botanists (Hooker 1880; Pitard 1923; Deb 1996; Lo 1999; Wright 1999; Chen and Taylor 2011). However, a recent molecular phylogenetic study has shown that *Myrioneuron* and *Mycetia* are intermixed, indicating that they do not form separate monophyletic groups (Ginter et al. 2015). Consequently, the two taxa have been merged into a single monophyletic genus, with *Mycetia* being accepted as the correct generic name (Ginter et al. 2015; Song and Xu 2016; Xu et al. 2016). The broadly delimited *Mycetia* is a medium-sized group comprising approximately 54 species distributed from South China through tropical Asia to the Northwest Pacific (Hassler 2025). It can be readily distinguished from the other five genera within the tribe Argostemmateae by its indehiscent berry-like fruits, which turn from green to white at maturity (Rydin et al. 2009; Ginter et al. 2015; Yan et al. 2016). Additionally, the straw-yellow corky bark on the upper part of the plant and calyx lobes bearing glands can serve as diagnostic features for identifying *Mycetia* species (Yan et al. 2016).

Despite the distinct characteristics of *Mycetia*, a comprehensive taxonomic revision of the genus has not been completed (Chaturvedi et al. 2011; Yan et al. 2016; Bajan et al. 2017; Tandang et al. 2019; Vu et al. 2020; Bora et al. 2025). Within the genus, the position of the inflorescence is considered one of the most important characters for infrageneric classification (Ridley 1923; Fukuoka 1989; Yan et al. 2016). Yan et al. (2016) recognized 13 *Mycetia* species with cauliflorous inflorescences and provided a key to these species for the first time. In China, “Flora Reipublicae Popularis Sinicae” (Lo 1999) and “Flora of China” (Chen and Taylor 2011) recorded 15 *Mycetia* species and four *Myrioneuron* species. Recently, a new species, *M. fangii* K.J. Yan & Z.Q. Song, was described from Guangxi (Yan et al. 2016), while *M. coriacea* (Dunn) Merr. was transferred by Song and Xu (2016) to *Foonchewia* R.J. Wang of the tribe Foonchewieae R.J. Wang. As a result, a total of 19 species are now recorded in China, including four species with cauliflorous inflorescences.

During a collecting trip to Yunnan, South China, we discovered an interesting cauliflorous *Mycetia* species in a limestone forest. Through detailed morphological comparisons with relevant species and phylogenetic analyses of five plastid DNA regions, we confirmed that this plant represents a previously undescribed species. Here, we provide a full description and name for this new species.

## ﻿Materials and methods

### ﻿Morphological studies

We conducted morphological studies on all available specimens of *Mycetia* housed in the herbaria BKF, BM, BO, CANT, GXMI, HITBC, IBK, IBSC, K, E, NY, MO, US, PE, and SYS by visiting these herbaria. We also examined the images of some specimens deposited in the herbaria A, BISH, GH, L, MICH, SING, and U. Acronyms for these herbaria are consistent with the Index Herbariorum (Thiers 2025). Over the past four years, we have conducted extensive field investigations and observed the morphological characteristics of most Chinese *Mycetia* species.

### ﻿Conservation status assessment

Based on the IUCN (2024) criteria, we conducted a preliminary evaluation of the conservation status of the new species described here.

### ﻿Phylogenetic analyses

To elucidate the phylogenetic relationships of the new species with other taxa within the tribe Argostemmateae, we reconstructed a molecular phylogeny of the tribe using five plastid DNA regions (*atpB-rbcL*, *ndhF*, *rbcL*, *rps16*, and *trnTF*). This analysis was based on the framework established by a previous study (Rydin et al. 2009). Our dataset included 42 species from eight genera, covering all six genera of Argostemmateae. Notably, *Mycetia* was represented by 35 samples from 25 species, with 14 species newly sequenced in this study (see Suppl. material 1). The remaining sequences were sourced from GenBank (www.ncbi.nlm.nih.gov/genbank).

For the new samples, total DNA was extracted from silica gel-dried leaves using a modified CTAB method (Doyle and Doyle 1987). Plastid sequences were obtained using a genome skimming approach (Zeng et al. 2018). Paired-end (PE) sequencing was performed on an Illumina HiSeq X Ten instrument at the Beijing Genomics Institute (BGI) in Wuhan, China. Subsequently, the sequences were assembled using GetOrganelle (Jin et al. 2020), annotated by PGA (Qu et al. 2019), and checked with Geneious v11.0.4 (Kearse et al. 2012). For annotation, *Dunnia sinensis* Tutcher (GenBank number: MN883829) was used as the reference.

Each of the five plastid DNA matrices was individually aligned using MAFFT v7.490 (Katoh and Standley 2013) with the L-INS-i algorithm. The alignments were subsequently trimmed using trimAl v1.5 (Capella-Gutiérrez et al. 2009) with the “automated1” setting to eliminate poorly aligned or unreliable regions. In each trimmed alignment, gaps (“-”) at the leading and trailing positions were coded as missing data (“?”). The plastid regions were then concatenated into a combined dataset (Suppl. material 2), which contained 6,687 columns and 496 parsimony-informative sites.

Maximum likelihood (ML) analysis was performed using IQ-TREE v3.0.1 (Nguyen et al. 2015) to reconstruct the phylogenetic tree. In IQ-TREE, the best substitution model was identified using ModelFinder (Kalyaanamoorthy et al. 2017) with the parameter “-m MFP” according to the Bayesian information criterion (BIC), and the TVM+F+R2 model was chosen. To assess the reliability of the phylogenetic tree, we used the Shimodaira–Hasegawa (SH-aLRT) approximate likelihood ratio test and the ultrafast bootstrap (UFboot) approximation, with parameters set at “-alrt 1000 -bb 1000.” Clades were considered well supported if they exhibited an SH-aLRT value of 80% or greater and a UFboot value of 95% or greater.

## ﻿Results

### ﻿Morphological studies

**Figure 1.**
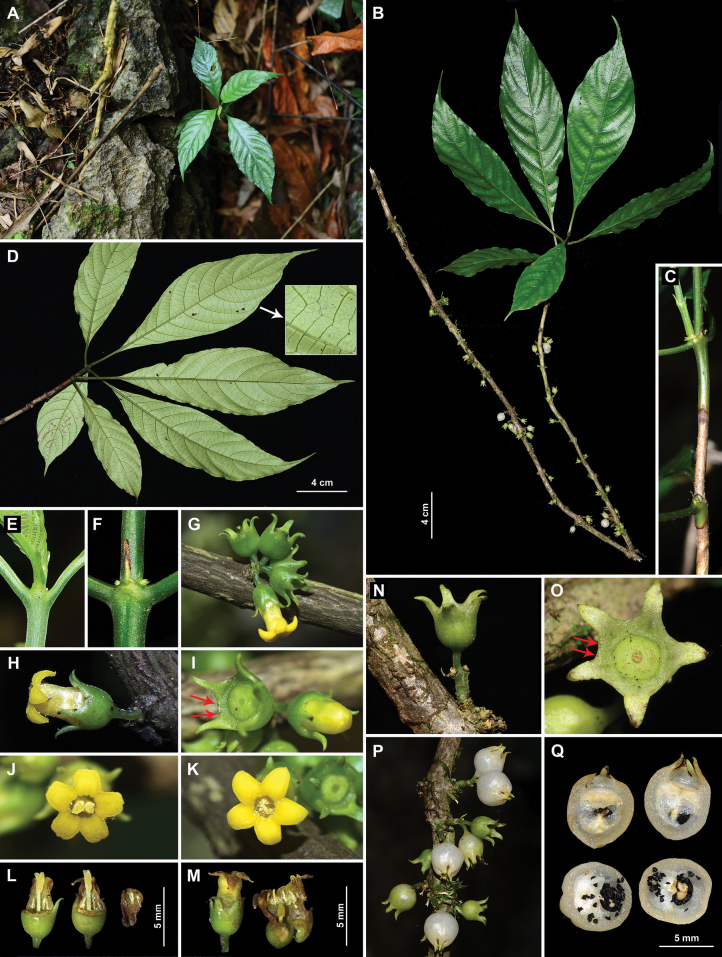
*Mycetia saxicola* Z.Q. Song & D.X. Xu. A. Habitat; B. Individual plant with cauliflorous infrutescences; C. Part of stem, bark green at the apical part and turning straw-yellow and corky at the upper part; D. Leaves in abaxial view; E. Uppermost stipule pair; F. Stipule in the upper part of the plant; G. Cauliflorous inflorescence; H. Single flower in lateral view; I. Calyx, arrows showing glands on the sinuses; J. Long-styled flower; K. Short-styled flower; L. Long-styled flowers, corolla partly removed; M. Short-styled flowers, corolla longitudinally slit on the right flower; N. Immature fruit in lateral view; O. Calyx, arrows showing glands on the sinuses; P. Cauliflorous infrutescences with immature and mature fruits; Q. Longitudinally (upper) and transversely (lower) opened mature fruits with numerous black seeds. Photos by Z.Q. Song (A–F, N–Q), B.Y. Zhang (H), and X.K. Xiong (G, I–M.).

Our morphological study revealed that the plant species discovered in limestone forests in Yunnan exhibits a suite of highly distinctive features. These include a limestone habitat (Fig. 1A), subcoriaceous leaves (Fig. 1B, D), straw-yellow corky bark on the upper part of the plant (Fig. 1C), linear stipules (Fig. 1E, F), cauliflorous inflorescences (Fig. 1B, G), a very short peduncle (1–2 mm long or absent; Fig. 1B, G, N), yellow heterodistylous flowers (long-styled flowers: Fig. 1J, L; short-styled flowers: Fig. 1K, M), very short pedicels (ca. 1.5 mm long; Fig. 1B, G, H, N, P), a short corolla tube (ca. 3.5 mm long; Fig. 1G, H, L, M), short calyx lobes bearing two sessile deciduous glands on each sinus (Fig. 1I, O), and 2-chambered berry-like fruits with numerous small, black seeds (Fig. 1Q). These features collectively indicate that this species is a unique member of the genus *Mycetia*. The cauliflorous nature and limestone habitat are the most significant features of this species. Comparisons of the species with two related species are documented in Table 1.

**Table 1.** Comparison between *Mycetia saxicola*, *M. fangii*, and *M. brevisepala*.

Character	*Mycetia saxicola*	*Mycetia fangii*	*Mycetia brevisepala*
Petiole	1.5–4 cm long	1–3(–5) cm long	0.2–1 cm long
Leaf texture	stiff chartaceous or subleathery	membranous to chartaceous	thinly leathery to papery
Leaf blade	9–22 × 3–8 cm	(4–)10–23 × (1–)3–6 cm	6–18 × 2.5–6 cm
Leaf shape	narrow elliptic or lanceolate, cuneate at base, gradually long acuminate at the apex	narrow elliptic or lanceolate, cuneate at base, gradually long acuminate at the apex	elliptic-oblong, elliptic, obovate, or oblong-lanceolate, base cuneate to obtuse, apex acuminate
Leaf hairiness	glabrous on both surfaces	glabrous on both surfaces	adaxially glabrous, abaxially glabrescent or densely puberulent to hispidulous on principal veins
Leaf lateral nerves	9–11 pairs	8–12 pairs	7–12 pairs
Stipule shape	linear, acute at the apex	ovate to suborbicular, obtuse at the apex	narrowly triangular, acute at the apex
Stipule size	4–6 × 1–2 mm	5–15 × 3–10 mm	3–6 mm × 1–2 mm
Stipule texture	subscarious	foliaceous	subscarious
Inflorescence position	cauliflorous	cauliflorous and axillary	terminal and cauliflorous
Peduncle length	1–2 mm long	7–14 mm long	10–20 mm long
Bracts and bracteoles	lanceolate, ca. 1 mm long	lanceolate or ovate, 1–2 mm long	narrowly triangular to lanceolate, 1–3 mm long
Pedicel length	ca. 1.5 mm long	4–8(–10) mm long	9–20 mm long
Flower number	usually less than 6	usually less than 10	9–25
Calyx lobes	deltoid-lanceolate, ca. 1.5 mm long, ca. 1 mm wide at base	deltoid-lanceolate, ca. 2 mm long, ca. 1 mm wide at base	triangular to narrowly triangular, 0.8–1 mm long
Calyx glands	two sessile glands in each sinus	one short gland in each sinus, 0.4–0.6 mm long	two sessile glands in each sinus
Corolla tube	ca. 3–4 mm long	ca. 5 mm long	ca. 5 mm long
Corolla lobes	deltoid-ovate, acute, ca. 1.5 mm long, recurved	deltoid-ovate, acute, ca. 1.5 mm long, recurved	deltoid-ovate, acute, recurved
Corolla hairiness	subglabrous outside	glabrous outside	glabrous outside
Fruit size	6–7 mm in diameter	6–7 mm in diameter	3.5–4 mm in diameter
Flowering	August to September	July to October	August to September
Fruiting	September to February	October to December	December
Habitat	limestone forest	non-limestone forest	non-limestone forest
Distribution	China (Yunnan)	China (Guangxi)	China (Yunnan) and northern Vietnam

**Figure 2.**
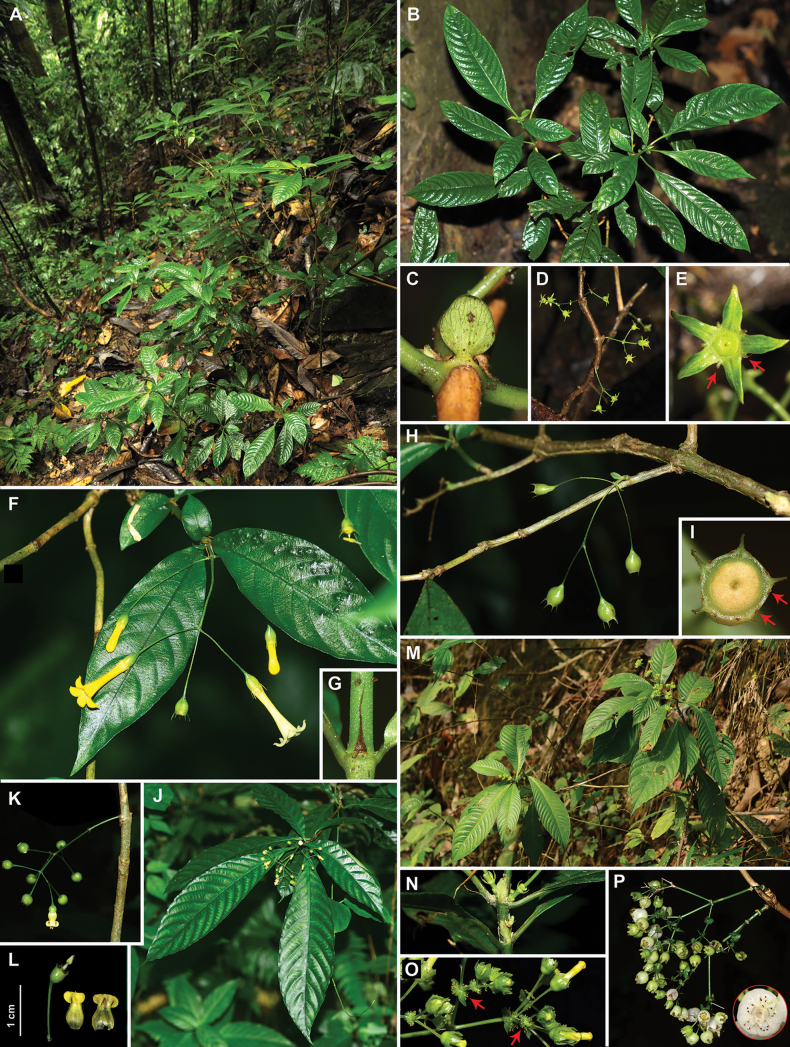
*Mycetia* species exhibiting cauliflory. A–E. *Mycetia fangii* K.J. Yan & Z.Q. Song; F–I. *Mycetia gracilis* Craib; J–L. *Mycetia brevisepala* H.S. Lo; M–P. *Mycetia yunnanica* H.S. Lo. A. Habitat; B. Leaves in adaxial view; C. Stipule; D. Cauliflorous infrutescences; E. Calyx, arrows showing glands on the sinuses; F. Branch with terminal inflorescence; G. Stipule; H. Cauliflorous inflorescence; I. Calyx, arrows showing glands on the sinuses; J. Branch with terminal inflorescence; K. Cauliflorous inflorescence; L. Dissection of flower; M. Habit; N. Stipules; O. Part of inflorescence, arrows showing glands on the bracts; P. Pseudo-axillary infrutescence, showing glands on the fruit calyx in the red circle. Photos by Z.Q. Song.

**Figure 3.**
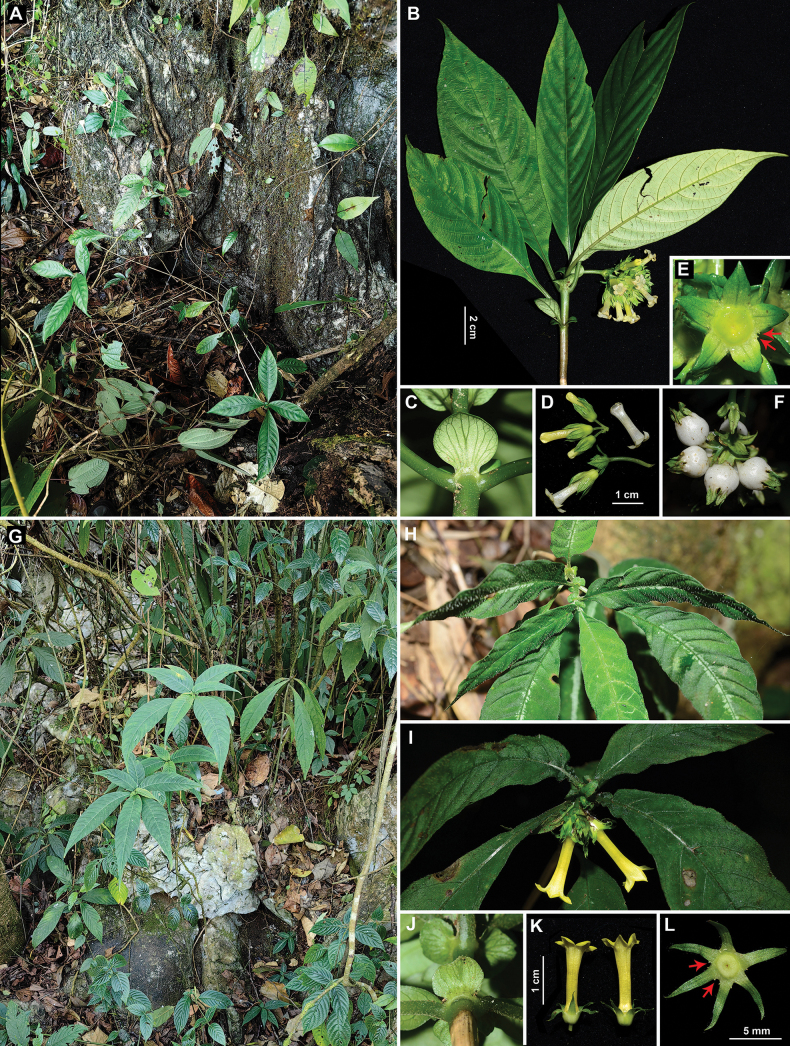
Two *Mycetia* species adapted to limestone habitat. A–F. *Mycetia macrocarpa* F.C. How ex H.S. Lo; G–L. *Mycetia anlongensis* H.S. Lo. A. Habitat; B. Individual with terminal inflorescence; C. Stipule; D. Flowers; E. Calyx, arrows showing glands on the sinuses; F. White berry-like fruits; G. Habitat; H. Membranous leaves covered with villosulous hairs; I. Terminal inflorescence; J. Stipule; K. Flowers; L. Calyx, arrows showing glands on the sinuses. Photos by Z.Q. Song.

We compared this taxon with all other congeneric species, particularly those with cauliflorous inflorescences and those growing in limestone habitats, by examining relevant literature, studying specimens, and observing living plants. Among these species, four Chinese species with cauliflory are illustrated in Fig. 2, including *M. fangii* (Fig. 2A–E), *M. gracilis* Craib (Fig. 2F–I), *M. brevisepala* H.S. Lo (Fig. 2J–L), and *M. yunnanica* H.S. Lo (Fig. 2M–P). Two species adapted to limestone habitats are illustrated in Fig. 3, including *M. macrocarpa* F.C. How ex H.S. Lo (Fig. 3A–F) and *M. anlongensis* H.S. Lo (Fig. 3G–L). These morphological studies have enabled us to confirm that the newly collected taxon represents a previously undescribed species, which we have named *Mycetia saxicola* Z.Q. Song & D.X. Xu.

### ﻿Phylogenetic relationships

**Figure 4.**
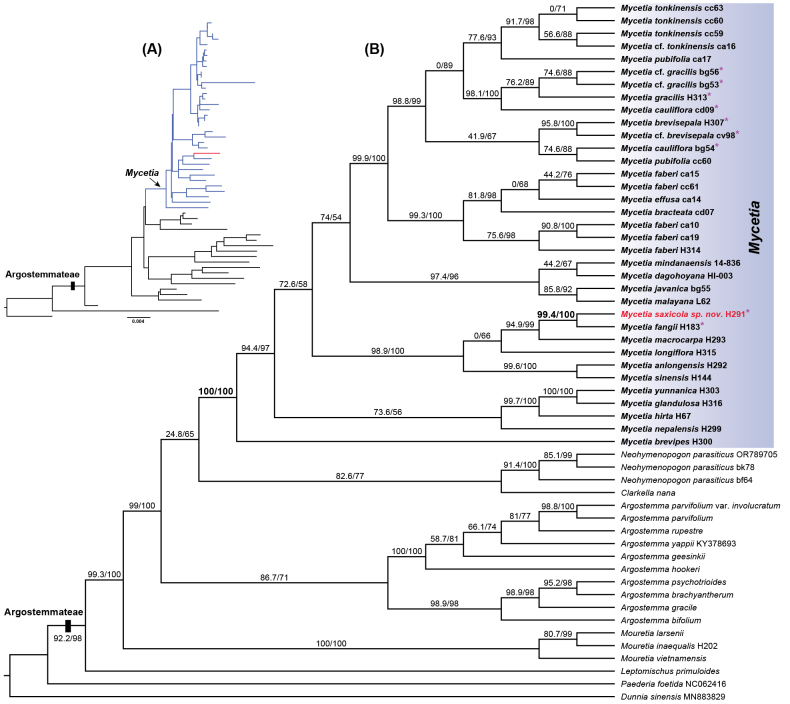
Maximum likelihood tree of the tribe Argostemmateae based on the combined dataset of five plastid markers (*atpB-rbcL*, *ndhF*, *rbcL*, *rps16*, and *trnTF*) using the software IQ-TREE v3.0.1. A. Phylogram with tip labels removed, blue lines showing the species of *Mycetia* and red line showing the new species described here; B. Cladogram with transformed branches. Numbers near branches are SH-aLRT and ultrafast bootstrap (UFBoot) support values (i.e., SH-aLRT/UFBoot). Purple stars behind the samples indicate species with cauliflorous inflorescences.

The phylogenetic tree derived from maximum likelihood (ML) analyses of the combined dataset of five plastid DNA regions (*atpB-rbcL*, *ndhF*, *rbcL*, *rps16*, and *trnTF*) is shown in Fig. 4. The species of the tribe Argostemmateae formed a strongly supported monophyletic group (SH-aLRT = 92.2%, UFBoot = 98%). Within Argostemmateae, four genera with multiple samples (*Argostemma*, *Mouretia*, *Neohymenopogon*, and *Mycetia*) were also found to be monophyletic. *Leptomischus* was shown to be sister to the other five genera. Within the genus *Mycetia*, the single species *Mycetia brevipes* F.C. How ex H.S. Lo, endemic to northwestern Yunnan, was revealed to be sister to all other congeneric species. Notably, the new species described here, *Mycetia saxicola*, was found to be most closely related to *Mycetia fangii*, with high support values (SH-aLRT = 99.4%, UFBoot = 100%).

## ﻿Discussion

### ﻿Phylogenetic relationships

Our molecular phylogenetic analysis based on plastid data elucidated the phylogenetic relationships of all six genera of the tribe Argostemmateae. *Leptomischus* and *Mouretia* were supported as successive sisters to the other four genera. This finding is similar to the results of three previous studies that utilized plastid data (Rydin et al. 2009; Ginter et al. 2015; Villanueva et al. 2016). These three studies, which did not include species of *Leptomischus* and *Clarkella*, found *Mouretia* to be the sister genus to the other three genera. However, our plastid-based results (Fig. 4) are in contrast to the recent findings from analyses of 353 nuclear genes by Thureborn et al. (2022), which did not sample any species of *Leptomischus* and revealed that *Mouretia* is most closely related to *Mycetia*, with *Argostemma* being the sister genus to the other four sampled genera.

For the genus *Mycetia*, we successfully obtained DNA samples from 25 species (approximately 46% of the whole genus), including those previously classified under *Myrioneuron* [e.g., *Myrioneuron faberi* Hemsl. ex F.B. Forbes & Hemsl., now recognized as *Mycetia faberi* (Hemsl. ex F.B. Forbes & Hemsl.) Razafim. & B. Bremer; Ginter et al. 2015]. Our analysis confirmed that *Mycetia* and *Myrioneuron* together formed a well-supported monophyletic group, thereby supporting the prior inclusion of *Myrioneuron* within *Mycetia* (Ginter et al. 2015). Within the broadened *Mycetia*, we discovered that the Himalayan species *M. brevipes* is the sister species to all other sampled congeners. Moreover, our molecular phylogenetic analyses distinctly highlighted the close relationship between *M. fangii* and the newly described species *M. saxicola*, the former being endemic to Guangxi Province, China (Yan et al. 2016).

### ﻿Morphological comparisons

Morphologically, the new species *Mycetia saxicola* closely resembles *M. fangii* in several key features, including the cauliflorous inflorescences, few flowers per inflorescence (usually less than 10), eglandular bracts and bracteoles, small flowers (about 5–8 mm long), and calyx lobes much shorter than the corolla. However, *M. saxicola* can be readily distinguished from *M. fangii* by its limestone habitat (Fig. 1A; vs. non-limestone habitat, Fig. 2A), subcoriaceous leaves (Fig. 1B; vs. membranous to chartaceous, Fig. 2B), linear stipules (Fig. 1E, F; vs. ovate to suborbicular, Fig. 2C), very short floral pedicels (ca. 1.5 mm long; Fig. 1L, M; vs. 4–10 mm long, Fig. 2D), and calyx bearing two sessile deciduous glands on each sinus (Fig. 1I, O; vs. calyx bearing one 0.4–0.6 mm long gland in each sinus, Fig. 2E).

Regarding the position of the inflorescence, Robbrecht (1988) identified four types within the tropical woody Rubiaceae: terminal, axillary, pseudo-axillary, and cauliflorous. Notably, the cauliflorous inflorescence is considered rare in the family. Yan et al. (2016) documented 13 *Mycetia* species exhibiting cauliflory worldwide. Different from all these species, *M. saxicola* has a very short peduncle (1–2 mm long or absent), short floral pedicels (ca. 1.5 mm long), and a short corolla tube (ca. 3.5 mm long). In contrast, the other species with cauliflory within *Mycetia* have a long peduncle (e.g., up to 7 cm long in *M. basiflora* Puff, 3 cm long in *M. yunnanica* H.S. Lo, and 1–2 cm long in *M. brevisepala* H.S. Lo), long floral pedicels (e.g., 9–15 mm long in *M. gracilis* Craib, 6–27 mm long in *M. cauliflora* Reinw., and 4–10 mm long in *M. fangii*), or an 8 mm or longer corolla tube [e.g., *M. mukerjiana* Deb & R.M. Dutta, *M. radiciflora* (C.B. Clarke) Airy Shaw, *M. brachybotrys* Merr., *M. flava* (Ridl.) Ridl., and *M. fasciculata* (Blume) Blume ex Korth.]. Among these, several species also possess terminal inflorescences in addition to their cauliflorous ones, such as *M. cauliflora*, *M. gracilis* (Fig. 2F, H), and *M. brevisepala* (Fig. 2J, K). In fact, terminal inflorescences are more common in these species. Field observations revealed that certain species develop pseudo-axillary infrutescences (not truly cauliflorous), which are derived from terminal inflorescences. This occurs when a new shoot emerges from the axil of the most apical leaf following flowering (Robbrecht 1988), as seen in *M. yunnanica* (Fig. 2M, P). These pseudo-axillary infrutescences were previously misinterpreted as cauliflorous. In the molecular analysis, our phylogenetic results indicate that the cauliflorous inflorescence likely evolved independently at least twice (see Fig. 4). To date, only two Chinese species, *M. fangii* and the newly described *M. saxicola*, lack terminal inflorescences and have only cauliflorous ones. This shared feature further supports their close phylogenetic relationship.

In their native habitat, *Mycetia* species are typically found in dense evergreen forests and moist environments, particularly along stream banks (Fukuoka 1989; Lo 1999; Chen and Taylor 2011; Yan et al. 2016; Bajan et al. 2017; Bora et al. 2025). Only a few species are adapted to drought-resistant limestone habitat, including *M. macrocarpa* (Fig. 3A) and *M. anlongensis* (Fig. 3G). Both species are endemic to China, with *M. macrocarpa* occurring exclusively in limestone forests and *M. anlongensis* sometimes found in non-limestone forests. *Mycetia macrocarpa* exhibits subcoriaceous leaves similar to those of the new species *M. saxicola*. Our phylogenetic analysis shows a relatively close relationship between these two species, with *M. macrocarpa* being resolved as the sister species to the clade containing *M. saxicola* and *M. fangii* (see Fig. 4). However, *M. saxicola* can be easily distinguished from *M. macrocarpa* by its linear stipules (vs. suborbicular to broadly elliptic stipules; Fig. 3C), cauliflorous inflorescences (vs. terminal inflorescences; Fig. 3B), a very short peduncle (absent or 1–2 mm long vs. a 15–40 mm long peduncle; Fig. 3B), shorter calyx lobes (ca. 1.5 mm long vs. 5–6 mm long; Fig. 3D) with two sessile glands on each sinus (vs. two distinct glands; Fig. 3E), a shorter corolla tube (ca. 3.5 mm vs. 12–14 mm long; Fig. 3D), and slightly larger mature fruits (6–7 mm vs. 7–10 mm in diameter; Fig. 3F). In contrast, *M. anlongensis* has membranous leaves covered with villosulous hairs on both sides (Fig. 3H), suborbicular or elliptic stipules (Fig. 3J), terminal inflorescences, long calyx lobes (ca. 6 mm long; Fig. 3L) with two distinct glands (Fig. 3L), and a long corolla tube (10–17 mm long; Fig. 3K), all of which are clearly distinct from those of *M. saxicola*.

### ﻿Taxonomic treatment

#### ﻿ Mycetia saxicola

Taxon classificationPlantaeGentianalesRubiaceae

Z.Q.Song & D.X.Xu sp. nov.

1C123B0B-5470-5C72-8B4F-89085849CB4D

urn:lsid:ipni.org:names:77372984-1

Figs 1, 5 岩生腺萼木

##### Type.

China. Yunnan Province, Malipo County, Tianbo Town, Yaowanggu, 23.022556, 104.825471, in limestone hills, elev. 1100 m, 27 November 2023, *Z.Q. Song & B.Y. Zhang JZ20231427* (holotype: IBSC1083200; Suppl. material 3).

##### Description.

***Shrubs***, 0.5–2 m tall, sometimes with little-branched; bark straw-yellow and corky at the upper parts, brown at the lower parts, but green at the apical parts; branches mostly ca. 4 mm in diameter. ***Leaves*** simple, opposite; petiole 1.5–4 cm, glabrous; blade 9–22 × 3–8 cm, narrow elliptic or lanceolate, cuneate at base, gradually long acuminate at the apex, stiff chartaceous or subleathery, glabrous on both surfaces, discolourous, deep green above and much paler green below; secondary nerves 9–11 pairs, alternate, subparallel, markedly curved at the margin. ***Stipules*** interpetiolar, linear, 4–6 × 1–2 mm, green at first and then turning brown, often deciduous. ***Inflorescences*** usually less than 1 cm long, directly borne on the stem (i.e., cauliflorous), trichotomously cymose, with a very short (1–2 mm long) peduncle, few-flowered, usually less than 6 flowers, sometimes 2–4 flowers fasciculate directly on the stem (peduncle absent); bracts and bracteoles lanceolate, ca. 1 mm long, without glands, caducous; pedicels very short, ca. 1.5 mm long. ***Flowers*** bisexual, heterodistylous, ca. 5 mm long. ***Calyx*** nearly hemispheric, ca. 2 mm high, subglabrous; lobes 5, persistent, deltoid-lanceolate, ca. 1.5 mm long, ca. 1 mm wide at base, with two sessile glands each sinus; glands deciduous. ***Corolla*** yellow, turning whitish at the end of the blooming period, urceolate, slightly swollen at base; tube ca. 3–4 mm long, subglabrous outside, pilose at middle position inside; lobes deltoid-ovate, acute, ca. 1.5 mm long, recurved. ***Stamens*** 5, subsessile; anthers situated at the base of the corolla tube for the long-styled flowers and situated at the upper of the corolla tube for the short-styled flowers, oblong, dorsifixed, ca. 1 mm long. ***Ovary*** 2-celled, with numerous ovules in each cell; style simple, slender, ca. 1.8 mm long for the long-styled flowers, ca. 0.5 mm long for the short-styled flowers; stigma bifid, linear, ca. 1.8 mm long for the long-styled flowers, ca. 1.2 mm long for the short-styled flowers. ***Fruits*** 2-chambered, subglobose, green and 3–3.5 mm in diameter when immature, turning white and baccate when mature, ca. 6–7 mm in diameter, crown with persistent calyx lobes. ***Seeds*** numerous, angled, ca. 0.3–0.5 mm across, slightly brown at first and turning shiny black when mature; testa granular.

##### Phenology.

Flowering from August to September and fruiting from September to February.

##### Distribution and habitat.

**Figure 5.**
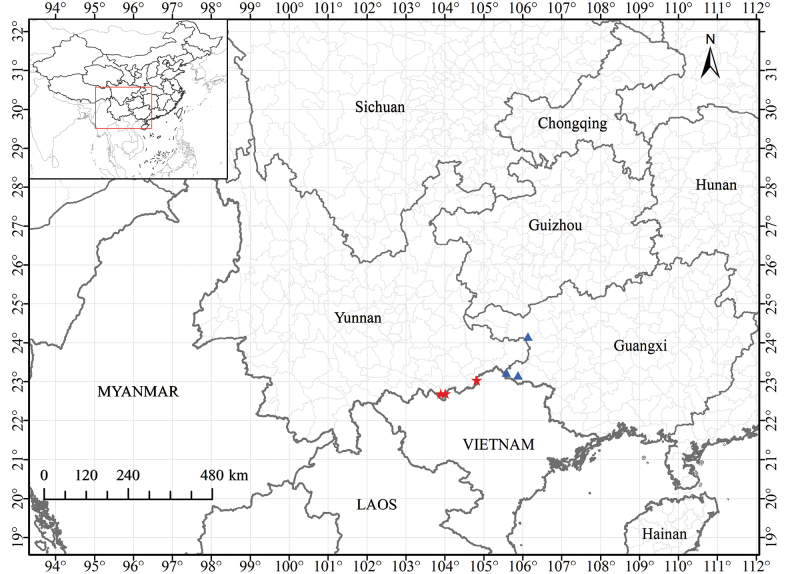
Distribution map of *Mycetia saxicola* Z.Q. Song & D.X. Xu (red stars) and its close relative *M. fangii* K.J. Yan & Z.Q. Song (blue triangles).

*Mycetia saxicola* is currently known from Malipo County and Hekou County, Yunnan Province, China (Fig. 5), and it grows in the understory of forests in limestone regions at 150–1100 m elevation. It is probable that this species will be found in northern Vietnam in the future.

##### Etymology.

The specific epithet *saxicola* is derived from the Latin *saxum* (rock) and the suffix -*cola* (dweller), emphasizing the limestone habitat, a distinctive and noteworthy feature of this new species.

##### Preliminary conservation status.

This new species is known from multiple localities in two counties in Yunnan Province, in both unprotected and protected limestone areas. Thus, the species may be considered a Near Endangered (NT) species under the IUCN categories (IUCN 2024).

##### Additional specimens examined.

China. Yunnan Province, Hekou County, Nanxi Town, Longyinchong, 22.685076°N, 104.019973°E, in limestone area, elev. 950 m, 5 September 2025, *B.Y. Zhang, Q.L. Yan & X.K. Xiong ZY202518* (IBSC); Yunnan Province, Hekou County, Nanxi Town, Maduoyi, 22.639901°N, 103.985103°E, in limestone area, elev. 323 m, 6 September 2025, *B.Y. Zhang, Q.L. Yan & X.K. Xiong ZY202520* (IBSC); Yunnan Province, Hekou County, Nanxi Town, Huayudong, 22.674730°N, 103.940051°E, in limestone area, elev. 141 m, 6 September 2025, *B.Y. Zhang, Q.L. Yan & X.K. Xiong ZY202521* (IBSC); Yunnan Province, Hekou County, Nanxi Town, Xiaoping, 22.681047°N, 103.903411°E, in limestone area, elev. 735 m, 7 September 2025, *B.Y. Zhang, Q.L. Yan & X.K. Xiong ZY202523* (IBSC).

## Supplementary Material

XML Treatment for Mycetia saxicola

## ﻿Additional information

### Use of AI

No use of AI was reported.

### Author ORCIDs

Zhuqiu Song https://orcid.org/0000-0002-7763-2431

Buyun Zhang https://orcid.org/0000-0003-1201-7638

Xiaokai Xiong https://orcid.org/0009-0009-8156-6391

Dongxian Xu https://orcid.org/0000-0002-7672-8300

### Data availability

All of the data that support the findings of this study are available in the main text or Supplementary Information.

## Supplementary materials

Supplementary material 1 Voucher information for the DNA samples in the present study Data type xlsx https://binary.pensoft.net/file/1486924This dataset is made available under the Open Database License (http://opendatacommons.org/licenses/odbl/1.0/). The Open Database License (ODbL) is a license agreement intended to allow users to freely share, modify, and use this Dataset while maintaining this same freedom for others, provided that the original source and author(s) are credited.Zhuqiu Song, Buyun Zhang, Xiaokai Xiong, Dongxian Xu

Supplementary material 2 Concatenated dataset of five plastid DNA regions (*atpB-rbcL*, *ndhF*, *rbcL*, *rps16*, and *trnTF*) Data type fasta https://binary.pensoft.net/file/1486925This dataset is made available under the Open Database License (http://opendatacommons.org/licenses/odbl/1.0/). The Open Database License (ODbL) is a license agreement intended to allow users to freely share, modify, and use this Dataset while maintaining this same freedom for others, provided that the original source and author(s) are credited.Zhuqiu Song, Buyun Zhang, Xiaokai Xiong, Dongxian Xu

Supplementary material 3 Holotype of *Mycetia saxicola* Z.Q.Song & D.X.Xu Data type jpg https://binary.pensoft.net/file/1486926This dataset is made available under the Open Database License (http://opendatacommons.org/licenses/odbl/1.0/). The Open Database License (ODbL) is a license agreement intended to allow users to freely share, modify, and use this Dataset while maintaining this same freedom for others, provided that the original source and author(s) are credited.Zhuqiu Song, Buyun Zhang, Xiaokai Xiong, Dongxian Xu
