# Supplementary figures and images for: ﻿Mycetia saxicola (Rubiaceae), a new species with cauliflory from limestone areas in Yunnan, China, supported by morphological and molecular data
Source: PhytoKeys. 2025 Dec 8;267:233–48. doi: 10.3897/phytokeys.267.175218 (PMC12706492; doi:10.3897/phytokeys.267.175218)

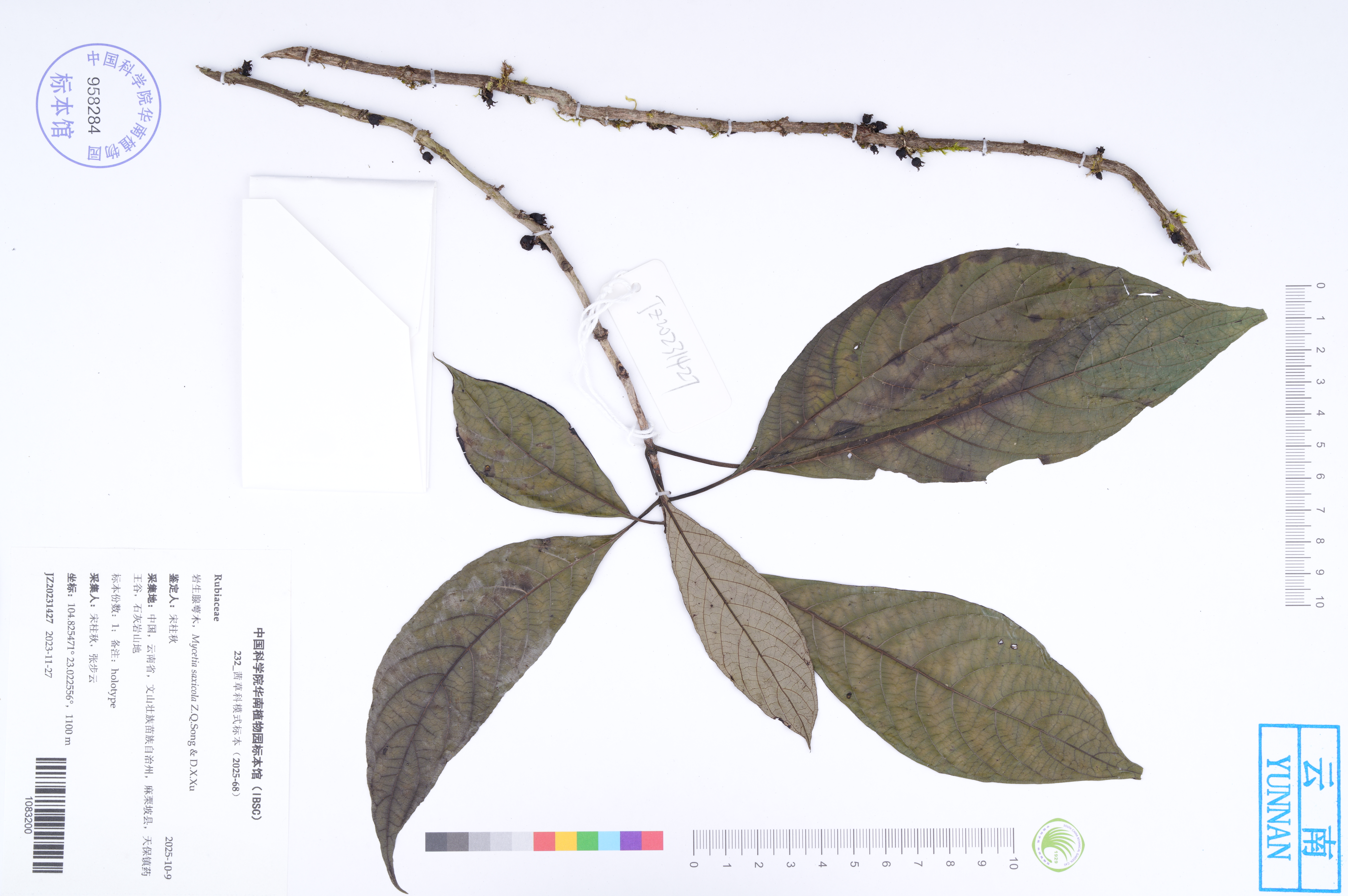

Supplement: Supplementary material 3 — Holotype of Mycetia saxicola Z.Q.Song & D.X.Xu [file phytokeys-267-233_article-175218__-s003.jpg]
